# Multidimensional metrics for estimating phage abundance, distribution, gene density, and sequence coverage in metagenomes

**DOI:** 10.3389/fmicb.2015.00381

**Published:** 2015-05-08

**Authors:** Ramy K. Aziz, Bhakti Dwivedi, Sajia Akhter, Mya Breitbart, Robert A. Edwards

**Affiliations:** ^1^Department of Computer Science, San Diego State UniversitySan Diego, CA, USA; ^2^Department of Microbiology and Immunology, Faculty of Pharmacy, Cairo UniversityCairo, Egypt; ^3^Computing, Environment, and Life Sciences, Argonne National LaboratoryArgonne, IL, USA; ^4^College of Marine Science, University of South Florida St. PetersburgSt. Petersburg, FL, USA

**Keywords:** virus, bacteriophage, genomics, metagenomics, ecology

## Abstract

Phages are the most abundant biological entities on Earth and play major ecological roles, yet the current sequenced phage genomes do not adequately represent their diversity, and little is known about the abundance and distribution of these sequenced genomes in nature. Although the study of phage ecology has benefited tremendously from the emergence of metagenomic sequencing, a systematic survey of phage genes and genomes in various ecosystems is still lacking, and fundamental questions about phage biology, lifestyle, and ecology remain unanswered. To address these questions and improve comparative analysis of phages in different metagenomes, we screened a core set of publicly available metagenomic samples for sequences related to completely sequenced phages using the web tool, Phage Eco-Locator. We then adopted and deployed an array of mathematical and statistical metrics for a multidimensional estimation of the abundance and distribution of phage genes and genomes in various ecosystems. Experiments using those metrics individually showed their usefulness in emphasizing the pervasive, yet uneven, distribution of known phage sequences in environmental metagenomes. Using these metrics in combination allowed us to resolve phage genomes into clusters that correlated with their genotypes and taxonomic classes as well as their ecological properties. We propose adding this set of metrics to current metaviromic analysis pipelines, where they can provide insight regarding phage mosaicism, habitat specificity, and evolution.

## Introduction

Viruses are the most abundant and diverse nucleic acid-based entities on Earth (Weinbauer, 2004; Edwards and Rohwer, 2005; Thurber, 2009). Their population densities are estimated to be 10^9^ per gram of soil (Williamson et al., 2005), 10^7^ per ml of seawater (Bergh et al., 1989; Wommack and Colwell, 2000), and 10^31^ planet-wide (Whitman et al., 1998). There are approximately 10 times as many viruses as the combined number of all cellular organisms, and most viruses are bacteriophages (phages), viruses that infect bacteria (Edwards and Rohwer, 2005).

Although phages play critical biological and ecological roles (Weinbauer, 2004; Abedon, 2009; Breitbart, 2012) and are the cornerstone of major molecular biology discoveries, the current number of completely sequenced phage genomes lags behind those of cellular organisms, and information about the abundance and distribution of these sequenced phage genomes in various ecosystems remains limited. A striking example of how little we know about phage abundance and distribution is that two prevalent phages with near-universal distribution in the oceans (Zhao et al., 2013) and human feces (Dutilh et al., 2014) were part of the unknown biological dark matter until only recently.

Traditional experimental strategies tend to underestimate phage diversity, mostly because culture-based methods miss the majority of phages. Furthermore, the actual abundance of phage nucleic acids in the environment is greater than that calculated from phage particle enumeration, since phage nucleic acids can be either packaged in free phage particles, or concealed as prophages within bacterial and archaeal genomes (Edwards and Rohwer, 2005; Angly et al., 2006). On the other hand, sequence-based strategies, notably the metagenomics technologies developed in the past decade (Breitbart et al., 2002; Breitbart and Rohwer, 2005), have revolutionized phage ecology (e.g., Breitbart et al., 2003; Angly et al., 2006; Thurber et al., 2009; Belcaid et al., 2010; Rodriguez-Brito et al., 2010; Swanson et al., 2011; Mizuno et al., 2013; Martinez Martinez et al., 2014). Despite those major advances, systematic surveys of phage genes and genomes in available metagenomes remain scarce partly because of the lack of well-established mathematical methods or metrics that define various aspects of phage distribution, abundance, and gene coverage.

Here we set out to define and deploy a set of metrics to better describe multiple dimensions of phage ecological properties. To this end, we implemented a scaffolding approach through the Phage Eco-Locator web-tool [URL: http://www.phantome.org/eco-locator (Aziz et al., 2011)], combined with a multidimensional set of metrics to enable a systematic analysis of phages in nature. To demonstrate these metrics and explore their significance, relevance, and applicability, this manuscript describes the abundance, ubiquity, diversity, and habitat-specificity of 588 completely sequenced viruses in 296 metagenomes from various ecosystems (Figure S1). The metrics described here can be used, individually or in combination, for the analysis of any set of metagenomes vs. any set of phages, regardless of the analysis platform, as long as the number of phage hits per metagenomic sample is available.

## Methods

### Input sequence data (Figure S1)

1. **Viral genomic data**. Viral genome sequences (582 phages, four of which contain three-segment genomes, i.e., three contigs each, as well as six archaeal viruses) were directly downloaded from the PhAnToMe database (URL: http://www.phantome.org/Downloads).
2. **Metagenomic data**. The 296 metagenomic data sets used for testing the methods consist of unassembled metagenomic sequences that had been originally annotated or re-annotated in the Metagenomics RAST server–version 3 (Meyer et al., 2008), then were cleaned up (Schmieder et al., 2010; Schmieder and Edwards, 2011a) or dereplicated (Schmieder and Edwards, 2011b) and deposited in MyMgDB (URL: http://edwards.sdsu.edu/cgi-bin/mymgdb/show.cgi). The sources of these metagenomic data sets and other metadata used in the analysis are provided in supporting online material (Table S1). Bacterial community structure in the same metagenomic data sets was analyzed by FOCUS (Silva et al., 2014).

### Phage Eco-Locator

Phage Eco-Locator (URL: http://www.phantome.org/eco-locator) is a Web interface, written in a combination of PERL, GnuPlot, and CGI scripts, that stores and visualizes precomputed tBLASTX (Altschul et al., 1997) results using dereplicated metagenomic DNA sequence reads as BLAST queries against a database of complete phage genomes (Aziz et al., 2011). For this study, a tBLASTX match to a phage sequence was considered significant if it had an *E*-value ≤ 10^−5^. The web tool allows examining matches with *E*-value threshold of 0.01 as well.

### Metrics describing phage abundance and distribution in ecosystems

As indicated in the Introduction section, this work was launched with the goal of defining and testing metrics that describe different aspects of phage ecological properties, through the interpretation of phage metagenomic recruitment plots, to compare the abundance and distribution of sequences from different phages in various metagenomes, and also compare different metagenomic samples based on their phage content and abundance.

**Figure 1:**
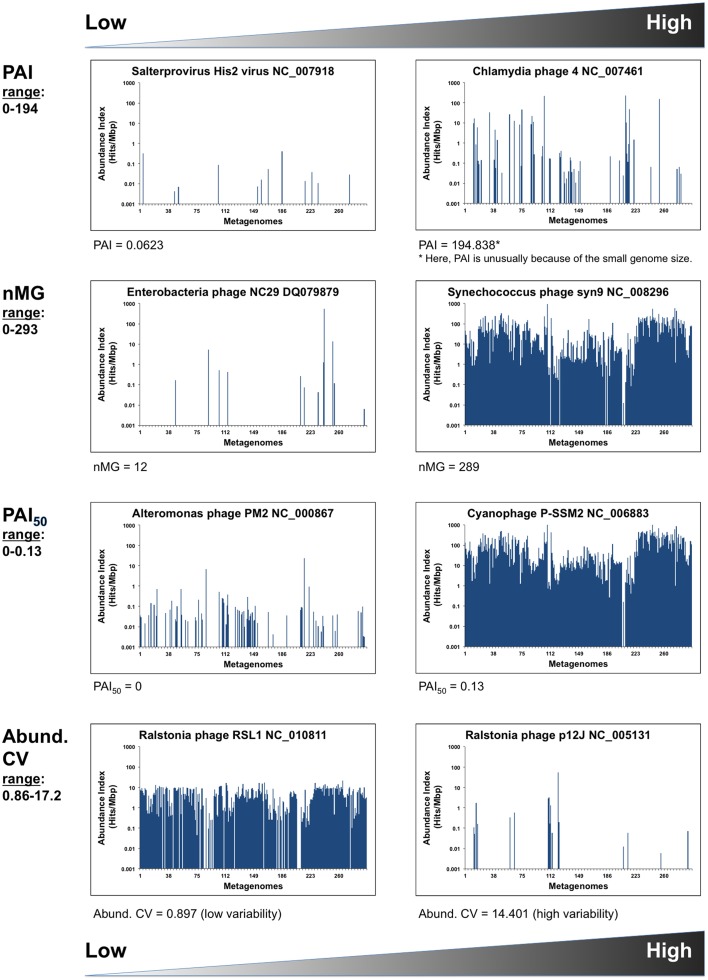
**Phage distribution metrics**. Inter-phage metrics and statistics quantifying different aspects of phage abundance and distribution in 296 metagenomic samples. Graphical examples show the phage genomes at the high and low ends of each parameter. X-axes represent the metagenomes (MG) listed in the same order as in Table S1 (i.e., grouped by environment). Y-axes are in logarithmic scales.

**Figure 2:**
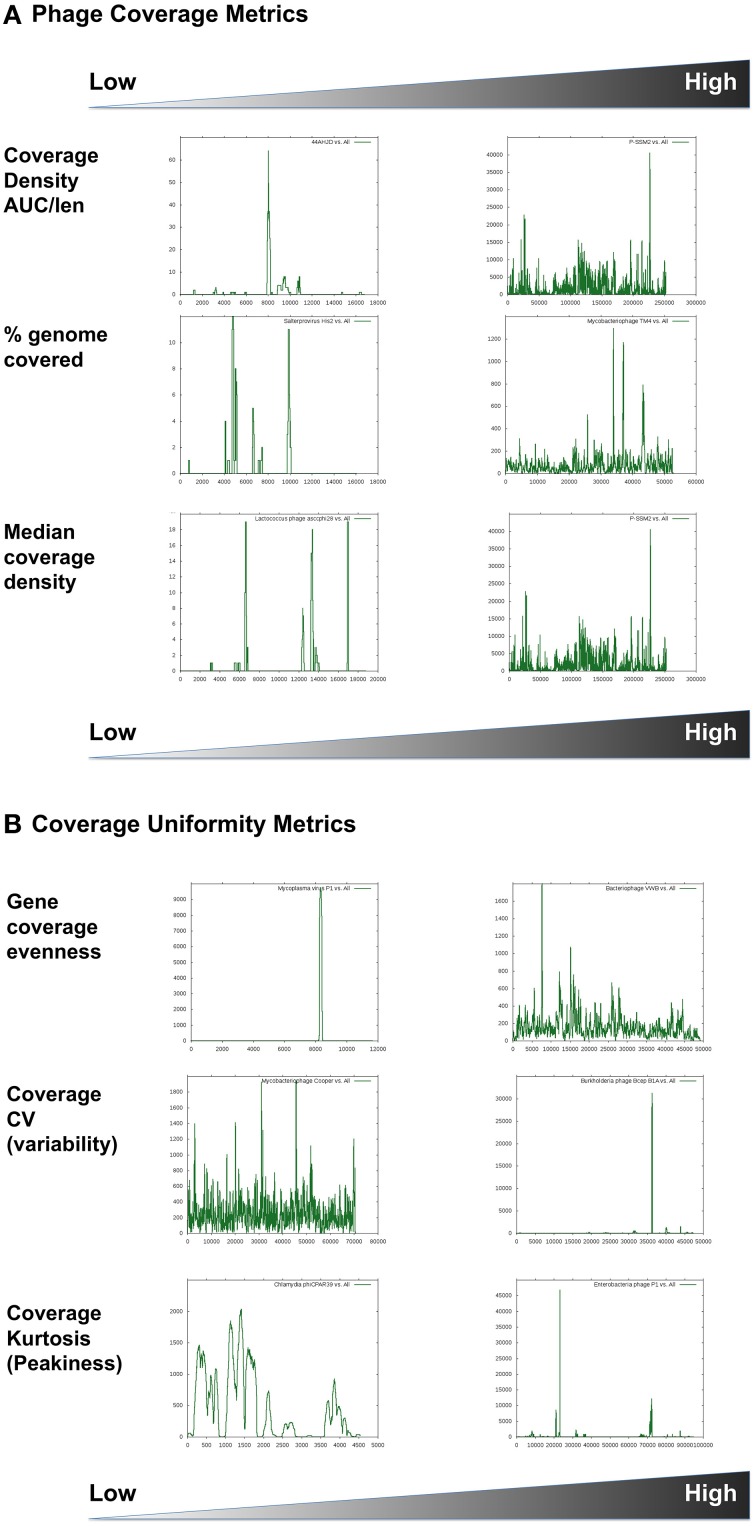
**Phage coverage metrics, including (A) density and (B) uniformity estimates**. Graphical examples show high and low ends of each parameter used. X-axes represent the genome coordinates while Y-axes represent number of hits to each nucleotide. Graphs are scaled differently. The coverage plots are for the following phages: **(A)** *Staphylococcus* phage 44AHJD compared to Cyanophage P-SSM2; Salterprovirus His2 virus compared to Mycobacteriophage TM4; *Lactococcus* phage asccphi28 compared to Cyanophage P-SSM2. **(B)** *Mycoplasma* virus P1 compared to Bacteriophage VWB; Mycobacterophage Cooper compared to *Burkholderia cenocepacia* phage BcepB1A; *Chlamydia* phage phiCPAR39 compared to Enterobacteria phage P1.

Those metrics fall into two major groups:

- Metagenome-level metrics: Metrics comparing different metagenomic data sets based on phage abundance and distribution (Table 1).
- Phage genome-level metrics:
  - Metrics that describe a specific phage's abundance and distribution (on the genome level) (Table 2, Figure 1).
  - Metrics that describe the pattern of abundance, distribution, and coverage of different genes or segments within a specific phage genome in metagenomic data sets (Table 3, Figure 2).

**(i) Metagenome-level metrics** (Table 1). The following metrics are defined to provide a comparison between different metagenomes based on the abundance and distribution of sequences similar to characterized phages that they contain.

**Table 1:** **Metrics used to describe and compare different metagenomes based on their phage content (metagenome-level metrics)**.

**Parameter**	**Definition/Calculation**	**Range**	**Significance/Interpretation/Limitations**
**IN A GIVEN METAGENOME Y**			
Abundance index (AI) of phage X	nHits of phage X/size of metagenome Y (Mbp)	0–1.244	This value describes the fraction of a metagenome library that matches a given phage genome. Dividing the number of sequence hits by the metagenome size (in millions of basepairs) permits comparison of different metagenomic samples.
Total AI	Σ nHits of a set of phages/size of metagenome Y (Mbp)	4.067–28.859	This value reflects the abundance of all sequences with similarity to phages in a metagenomic library. *Limitations*: sensitive to outlier AI values (contaminants, sequencing artifacts, unusually large number of hits), i.e., false positive hits of a single phage can artificially inflate this value.
Median AI (AI_50_)	AI of the 50th percentile phage genome	0–3.061	This value gives an indication of the abundance of sequences with similarity to phages within a metagenomic library and is less sensitive to outliers than Total AI; however, it may underestimate real differences between samples (e.g., if more than half of the phage genomes have no sequence similarities to a metagenomic library, AI_50_ will be zero regardless of whether the total abundance of the remaining phage genomes is high or low).
nPhages (richness)	Number of phage genomes which match at least one sequence read in metagenome Y	8–487	This value is a proxy for *richness* of phage types within the metagenomic sample. While this value may overestimate the number of phage types within the tested sample, it can be used to compare sequence diversity between the tested metagenomic samples.
Shannon Diversity Index	H = -Σ *p_i_* ln *p_i_* where *p_i_* is the proportion of sequence hits to the i^*th*^ phage genome relative to all phage genome hits within the metagenome	2.061–5.813	This value (Shannon, 1948) is an indication of the *diversity* of phage sequences within a metagenomic sample, but is not an accurate estimation of phage species diversity [which is beyond the focus of this paper and is to be calculated by other tools, e.g., PHACCS (Angly et al., 2005) or Shotgun UNIFRAC (Caporaso et al., 2011)].
Shannon E (evenness)	E = H/ln nPhages	0.008–0.258	This value describes the *evenness* of distribution of phage genomes. When Shannon *E*-value = 1, all genomes are equally represented; a Shannon *E*-value that is closer to zero reflects that an uneven distribution where some genomes are much more represented than others.

**Table 2:** **Metrics used to describe phage ecological features at the genome level**.

**Parameter**	**Definition/Calculation**	**Range**	**Significance/Interpretation/Limitations**
**PHAGE DISTRIBUTION METRICS (GENOME-LEVEL METRICS): FOR A GIVEN PHAGE X**			
Phage abundance index (PAI)	Σ AI of phage X (hits per Mbp)/length of phage X (in Kbp)	0–194.84	This value describes the abundance of a phage in a set of environments. Normalizing the AI of each phage genome to the genome length allows the comparison of different phages. This normalization is useful for most phages; however, it might artificially inflate PAI value if the phage genome is significantly smaller than the median genome size, which is ~41 Kbp (e.g., microviruses, with 4 Kbp genomes)
nMG	Number of metagenomes with hits to phage X	0–293	This value reflects the ubiquity of a particular phage genome. A high nMG suggests that a phage genome (or part of it) is universally distributed or cosmopolitan; a low nMG suggests that the phage is localized or ecologically limited (i.e., specific to one or a few habitats).
PAI_50_	Median AI of phage X in all tested metagenomes/length of phage X	0–0.13	This value is another indication of the abundance of a phage genome in different metagenomic samples and is less sensitive to outliers. It is also dependent on the ubiquity of a phage genome since PAI_50_ of phage genomes present in fewer than half samples, for example, will be zero, even if these genomes have a high PAI.
Abund. CV (Coefficient of variation)	StDev/Mean AI of phage X	0.86–17.20	This value reflects the spread or variation of AIs of a given phage among metagenomes. A large CV suggests that a phage genome has extreme AIs while a small CV suggests uniform AI values (but doesn't give information on their magnitude).

*Representative examples of each value are given in Figure 1*.

**Table 3:** **Metrics used to describe phage ecological features at the nucleotide level**.

**Parameter**	**Definition/Calculation**	**Range**	**Significance/Interpretation/Limitations**
**PHAGE COVERAGE METRICS (INTRA-PHAGE OR NUCLEOTIDE-LEVEL METRICS): FOR A GIVEN RECRUITMENT PLOT OF A PHAGE X**			
Coverage density (AUC/nNuc)	Area of a genome coverage plot (area-under-the curve) normalized to the total number of nucleotides in the phage genome.	0–2.920	This value is similar to the total abundance of a phage in all metagenomes; however, it also considers each nucleotide covered in the phage genome and not just the number of sequence reads that match that genome.
Density per metagenome (cumulative AUC/nMG)	Average overall phage density divided by the number of metagenomes.	126–1.71 × 10^6^	This value normalizes the coverage density to the number of metagenomes in which the phage genome is found. It differentiates between the densities of ubiquitous phages (high nMG) and that of habitat-specific phages (low nMG).
%genome covered	Fraction of the phage genome that matches at least one metagenomic sequence.	0–100%	This value reflects the homogeneity of overall phage coverage in metagenomes as well as the gaps in coverage. It marks areas within a phage genome that have not been matched in any metagenomic sample, but is magnitude-independent—thus does not show which areas of the genome are overrepresented. A %genome coverage of 40% means that combined uncovered gaps are 60%.
Gene coverage evenness	Adapted Shannon Evenness Index (Shannon E) of the coverage of phage genes. E = -Σ *p_i_* ln *p_i_* /nGenes where *p_i_* is the proportion of hits to the *i*^th^ gene to the sum of hits to all genes of phage X	0–0.92	This value reflects whether protein-encoding genes within a phage genome are equally represented relative to each other. A gene evenness of one means that all phage genes are equally represented (regardless of the magnitude of their coverage), while low evenness values suggest possible non-specific or cross-matching genes (i.e., parts or all of the phage genome is absent).
Coverage coefficient of variation (CV)	Standard deviation of coverage density/Mean coverage density (Coverage density = AUC/nNuc)	0.76–12.58	This value reflects the variation or spread of coverage along a phage genome. Typically a phage genome coverage plot with high CV has higher coverage values for certain parts of the genome and zero values for other parts.
Median coverage density	Median number of hits per nucleotide per phage	0–686	Less sensitive to extreme values, the median coverage density provides another indicator of the homogeneity of phage genome coverage in metagenomic samples.
Coverage kurtosis	Kurtosis equation: $\frac{\sum {\left(X-\mu \right)}^{4}}{N\sigma^{4}}-3$ where *X* is the value of each data point, *μ* is the sample mean, *σ* is the standard deviation, and *N* is the number of data points	0.02–423.12	Kurtosis is a statistical measure of uniformity or lack thereof within a frequency distribution curve. It is often used as a measure of skewness, bimodality, or “peakiness” of a distribution plot. It has been adopted here to reflect the irregularity of a phage coverage density plot. If a phage genome coverage plot has high kurtosis, this means that some areas of this genome have sharp coverage peaks while others have low or no coverage values. Negative kurtosis values reflect flatter coverage plots but do not provide information about the coverage magnitude.

*Representative examples of each value are given in Figure 2*.

First, *all* metagenomic sequence reads with significant tBLASTX hits to phage sequences were collected from Eco-Locator recruitment plots and stored for further calculations. Those values were counted and defined as nHits. Default significance thresholds were set at BLAST *E*-values of 10^−5^.

Next, an *abundance index* (AI) was calculated for each metagenome. For a given metagenome, the AI was defined as the number of hits to phage genomes (nHits) normalized to the metagenome size in millions of base pairs.

AI=nHits/metagenome size, Mbp

Subsequently, a *total abundance index* was defined for each metagenome to express the overall abundance of sequences with similarities to characterized phage genomes in that metagenome.

$$\begin{array}{l} \text{Total abundance index }(\text{of all phage genomes})\text{ per metagenome}\\ \;=\Sigma (\text{nHits}/\text{metagenome size},\text{ Mbp}) \end{array}$$

Because of the high variability of phage types in different ecosystems, the total AI defined above is highly sensitive to outliers, and thus the *median AI* of sequences with similarities to characterized phage genomes per metagenome was calculated as another useful value to compare metagenomes and reflect their phage content.

In addition to AI and median AI, which reflect phage-like metagenomic fragment counts, we also used some commonly used ecological biodiversity parameters such as richness, diversity, and evenness, described elsewhere (Shannon, 1948 disambiguated in Spellerberg and Fedor, 2003).

A full list of metagenome-level metrics, and the significance of each, is provided in Table 1.

**(ii) Phage genome-level metrics**.
  - **Inter-phage properties** (Table 2). For comparison of phage genomes, a *phage abundance index* (PAI) was defined for each phage and calculated as the number of metagenomic sequence fragments assignable to that phage genome normalized to the genome size

PAI=Σ AI/Phage genome length (Kbp)

Because PAI depends on summing up all available metagenomic sequences that are similar to a particular phage, this value reflects a phage's overall abundance in a set of ecosystems, but provides little information about the pattern of its distribution, since a very high PAI may be contributed by an overabundance in a small number of metagenomes (nMGs).

Instead, an estimation of the distribution of a certain phage in a given set of ecosystems may be expressed as a simple count of the nMGs with significant BLAST hits (*E*-value < 10^−5^) to a given phage genome. With a large nMGs from distinct ecosystems, nMG can be reliably used as a proxy for phage ubiquity in nature. In addition to counting metagenomes with hits to a given phage, we calculate the median PAI (PAI_50_), an estimator of both the abundance and ubiquity of that phage in nature (Table 2).

**Figure 3:**
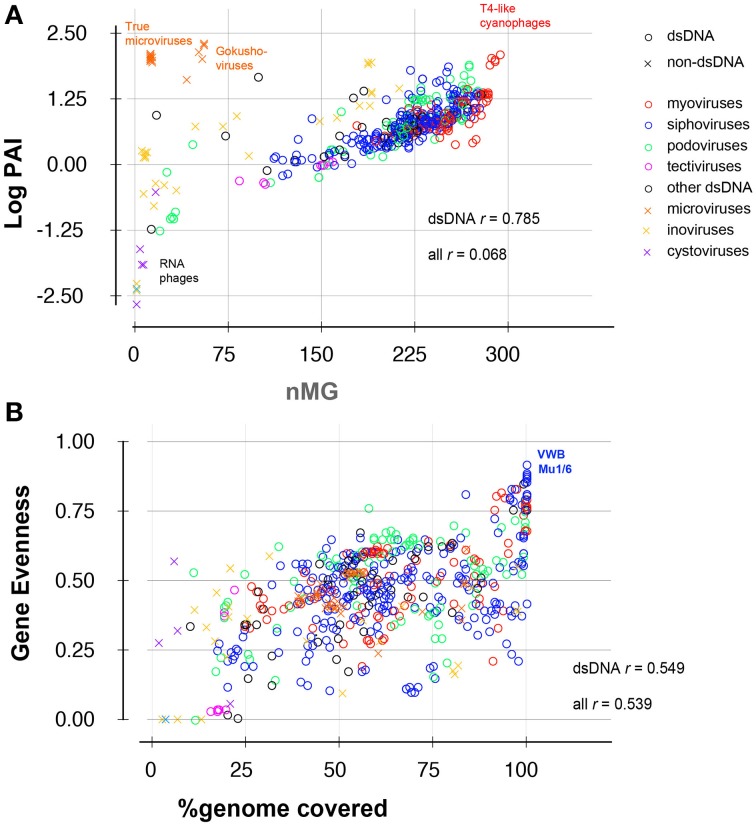
**Scatter plots showing correlation between (A) abundance and ubiquity or (B) gene evenness and % genome coverage of 588 viruses in 296 metagenomes**. Data points are labeled according to phage family (different colors), and nucleic acid content (circles: dsDNA phages; crosses: other phages, i.e., ssRNA, dsRNA, and ssDNA phages). Correlation coefficients (*r*) are shown for all phages and for dsDNA phages alone.

Combining PAI, PAI_50_, and nMG in comparisons between different phages provides a good multidimensional picture of phage distribution in nature, balancing abundance and ubiquity, as those two values do not necessarily correlate (Figure 3A). Those values, however, do not tell much about the uniformity of a phage's distribution among ecosystems. A phage with high PAI and low nMG is expected to have a highly variable distribution pattern in nature. This variability can be expressed as the *abundance coefficient of variation* (Abundance CV), representing the *data spread* of a phage genome's AI across metagenomic data sets, where CV is the standard deviation divided by the mean.

Abundance CV=σ AI/mean AI

(b) **Intra-phage properties** (Table 3). Fragment recruitment plots and genome coverage maps are quite popular in analyzing metagenomic data; yet, a wealth of information encoded within those plots remains unexplored. Phage Eco-Locator, like other common metavirome analysis tools, e.g., MG-RAST (Meyer et al., 2008) and MetaVir (Roux et al., 2011), displays fragment recruitment plots, in which each metagenomic fragment is aligned to the corresponding genomic segment, as well as genome coverage density plots, in which each metagenomic sequence is cumulatively plotted against a phage genome scaffold, at a nucleotide resolution.

Coverage density plots provide a quick visual estimate of phage sequence conservation and distribution in a given metagenome. However, these plots are often biased by the presence of short sequences that are highly abundant (e.g., short repeats indicative of transposons or insertion sequences). Several mathematical values are suggested here to estimate different features of sequence coverage along a phage scaffold (Table 3). For example, *coverage density* may be measured as the area under the curve (AUC) normalized to the genome length (in nucleotides). For a certain phage, the total (or cumulative) coverage density in a large set of metagenomes may be further normalized to the nMGs with hits to that phage. As with other metrics, coverage density or cumulative coverage density is sensitive to outliers. Thus, *median coverage density* can be used to reflect the homogeneity of phage genome coverage in metagenomic samples.

In addition to coverage density, recruitment can be described by the uniformity, regularity, or continuity of sequence coverage over the entire genome length. Uniformity may be measured in various ways. One way is to simply estimate the percentage of a phage genome that recruits metagenomic reads (with possible optimization of significance and alignment length thresholds). This value does not reflect the regularity or uniformity of the distribution, but indicates coverage gaps [sometimes referred to as metagenomic islands (Pasic et al., 2009; Mizuno et al., 2014)]. Other estimators of uniformity implemented in this study include the *spread* of a coverage plot (expressed as the coefficient of variation of coverage), *kurtosis* (a statistical value of a plot's uniformity), and an *adapted Shannon Evenness Index* applied to phage genes (explained in detail in Table 3). Examples of phage distribution and phage recruitment plots are provided in Figures 1, 2, and all raw data are provided in Table S2.

### Statistical analysis

For statistical analysis, DataDesk (Data Description Inc., Ithaca, NY; URL: http://www.datadesk.com) and the R software environment (URL: http://www.r-project.org) were used.

## Results

### Input data

Eco-Locator plots were generated for a core data set of 588 viral genomes and 296 metagenomes. Fragment-recruitment and coverage-density plots for each unassembled metagenome were generated and are publicly available (URL: http://www.phantome.org/eco-locator).

### Implementation and testing of metagenome-level metrics

Abundance values (expressed as total AIs) of sequences related to known phages showed an immense variation among different metagenomes, spanning several orders of magnitude (range = 4–28,859 hits /Mbp; mean = 1462.8 hits /Mbp; median = 1125 hits /Mbp). At the lower end, samples from human lungs, classically thought to be free of resident microbiota, had the smallest fraction of sequences similar to known phages and the lowest sequence diversity and richness as previously reported (Willner et al., 2009, 2012) (Table 4 and Table S1). Hypersaline samples also had low abundance indices, possibly resulting from the low number of completely sequenced viral sequences from these habitats (Table S1). At the other extreme, aquatic samples (both virus-enriched and microbial) contained the largest fraction of sequences similar to known phages. The microbial metagenome with highest phage AI was from the open ocean (Hydrostation S, Sargasso Sea, Bermuda), while the viral metagenome with highest phage AI was an estuary sample (Station 834, Chesapeake Bay Virioplankton) (Table 4 and Table S1). The sample with highest number of phage types (richness) was from a marine-derived lake in Antarctica, and those with highest phage sequence diversity (Shannon diversity) were human gut samples (Table 4).

**Table 4:** **Examples of the lowest and highest scoring metagenomes or phages according to different metrics**.

**Parameter**	**Low**	**High**
**METAGENOME-LEVEL METRICS:**		
Total AI	Lung samples (Table S1) (Values: 4.07–8.22)	Hydrostation S, Sargasso Sea, Bermuda (open ocean) (Value = 28.859)
Median AI (AI_50_)	Lung samples (Table S1) (Value = 0)	Chesapeake Bay, MD (estuary): Chesapeake Bay Virioplankton–Station 834 (Value = 3.061)
nPhages	Viral data from the human lung (Sample 109) Value = 8 phages	AntarcticaAquatic_5–Marine-derived lake (Value = 487 phages)
Shannon Diversity Index	Viral data from the human lung (Sample 109) Value = 2.061	Stool metagenome (sample 179) Value = 5.813
Shannon evenness E	GS051 Shotgun–Coral Reef Atoll–Polynesia Archipelagos–Rangirora Atoll–Fr. Polynesia (Value = 0.008)	Viral data from the human lung (sample 109) Value = 0.258
**PHAGE DISTRIBUTION METRICS:**		
Phage abundance index (PAI)	Eleven out of 17 RNA phages have zero values	*Chlamydia* phage 4 (ssDNA) Value = 194.84; Cyanophage P-SSM4 (dsDNA) Value = 109.856
PAI_50_	*Aeromonas* phage PM2 (Value = 0)	T4-like cyanophage P-SSM2 (Value = 0.13)
nMG	Eleven RNA viruses have zero values; *Pseudomonas* phi-6 (dsRNA, Value = 1); dsDNA: *Lactococcus* phage asccphi28 (Value = 20)	T4-like cyanophage P-SSM2 (Value = 293)
Abund. CV	Myoviridae Bacillus phage 0305phi8-36 (Value = 0.86)	Ralstonia phage P12 J (dsDNA, Value = 14.4), *Pseudomonas* phage phi-6 (dsRNA, Value = 17.2) and microviruses (ssDNA, Values > 16)
**WITHIN PHAGE COVERAGE/DENSITY METRICS (INTRAPHAGE PROPERTIES):**		
Coverage density	Levivirus Enterobacteria phage MS2 (ssRNA, Value = 0.04); *Staphylococcus* phage 44AHJD (dsDNA, Value = 1.03)	Coliphage phiX174 (ssDNA, Value = 2.920); T4-like cyanophage P-SSM2 (1.989)
Density per metagenome	Enterobacteria phage MS2 (ssDNA, Value = 126); *Lactococcus* phage asccphi28 (dsDNA, Value = 540.45)	T4-like cyanophage P-SSM2 (1.71 × 10^6^)
%genome covered	Salterprovirus His 2 (Value = 10%; lowest non-zero value for a dsDNA virus)	Mycobacteriophages Rosebush and Cooper (Value = 100%)
Gene coverage evenness	*Mycoplasma* virus P1 (lowest non-zero value = 0.003)	Bacteriophage VWB (Value = 0.918) and *Streptomyces* Mu1/6 (Value = 0.886)
Spread (CV)	Actinoplanes phage phiAsp (Value = 0.757)	*Burkholderia* phage BcepB1A (Value = 12.581)
Coverage kurtosis	*Chlamydia* phage phiCPAR39 (ssDNA, Value = 0.02); unclassified Picovirinae Actinomyces phage Av-1 (dsDNA, Value = 3.03)	Enterobacteria phage P1 (Value = 423.12)
Median density	*Lactococcus* phage Asccphi28 (among 254 phages with zero value)	T4-like cyanophage P-SSM2 (Value = 686)

*If the high end is not a dsDNA phage, the next highest/lowest dsDNA phage is also shown*.

An in-depth ecological analysis comparing all metagenomes or examining phage habitat-association is beyond the scope of this Methods Article; however, a glimpse at extreme values of each metric (Table 4) provides confidence in the methodology used because of its agreement with previous analyses performed on subsets of those data (Angly et al., 2006; Dinsdale et al., 2008; Willner et al., 2009) and because of some biologically relevant measurements (such as the low phage richness in lungs or the high phage diversity in stool samples).

### Implementation and testing of phage-level metrics

The most common statistics used in viral metagenomic studies rely on two key parameters: the relative abundance of phage-like sequences [defined here as PAI and referred to as depth in some other studies (Dutilh et al., 2014; Martinez Martinez et al., 2014)] and the nMGs in which a particular phage is represented (ubiquity or nMG) (e.g., Mizuno et al., 2013; Dutilh et al., 2014). These two statistics are undoubtedly useful, but are limited by the following: (i) we observed that plotting abundance and ubiquity successfully resolves classes of RNA or single-stranded DNA (ssDNA) viruses, yet these two metrics are partly interdependent among double-stranded DNA viruses (correlation index = 0.785, Figure 3A); (ii) abundance and ubiquity metrics quantitatively describe phage prevalence but do not describe the *pattern* of this prevalence (e.g., phage-ecosystem correlations or habitat-specificity); (iii) these values are sensitive to biases (for example, they may be strongly affected by the dominance of aquatic samples or human-associated samples in a data set). Accordingly, we implemented additional metrics to better assess the multidimensional nature of abundance and distribution of phage sequences as well as the intra-phage coverage density and evenness (detailed in Methods and Tables 2, 3). For example, we estimated the cross-habitat variation among AIs using the coefficient of variation (Abundance CV; Tables 2 and Table S2), which provides information on the homogeneity of distribution of phage sequences across metagenomes, and can differentiate between cosmopolitan and habitat-confined phages (Thurber, 2009).

Eleven RNA phages in our database were practically undetected. The absence of these RNA viruses is expected since the metagenomes analyzed consisted only of DNA and were not supposed to contain RNA contamination, and since there is little shared sequence similarity between RNA and DNA phage genes, as seen in the Phage Proteomic Tree (Rohwer and Edwards, 2002) and the Phage Population Network (Lima-Mendez et al., 2008).

**Figure 4:**
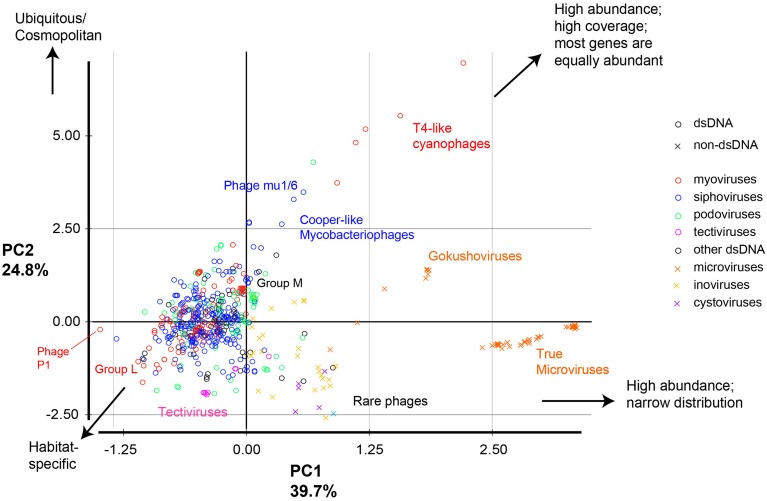
**Principal component analysis of phage genomes according to their ecological properties**. All phages were compared based on 11 metrics, then the 11 dimensions were reduced into two principal components that explain most of the variance. Circles represent dsDNA phages and x signs represent other types of phage genomes; colors represent different phage classes. Examples of phages and groups of phage discussed in the text are labeled.

Another important set of metrics implemented in this study describe the uniformity of sequence coverage within a phage genome, and thus help indicate whether phage abundance values represent presence of an entire related phage or result from the overabundance of specific conserved genes or tiny fractions of phage genomes. Of those values, the % sequence coverage in all metagenomes, for example, gives a good indication of the global distribution of phage modules, while the gene evenness parameter is an indicator of the covariation of different genes between different habitats (Figures 2A, 3B). Overall, more than a dozen metrics were used to describe the ecological and coverage properties of each phage genome (Table S2), 11 of which were selected (Tables 1, 2) and combined to separate all phage genomes based on two principal components that summarize the 11 dimensions and explain ~65% of the variance (Figure 4).

### Combining the multidimensional metrics separates phage genomes based on ecological parameters

Taken together, this combination of metrics allowed the separation of phages into distinct groups (Figure 4) according to their environmental abundance, distribution, and sequence coverage parameters. The most prominent groups are:

- Phages with high abundance, broad distribution, and low inter-sample variation (e.g., T4-like cyanophages, *Bordetella* phages, and *Streptomyces* Phage Mu1/6). This pattern indicates ubiquitous or near cosmopolitan phages, or—alternatively—phages highly similar to cosmopolitan phages.
- Phages with high abundance, broad distribution, but low gene evenness (e.g., Cooper-like mycobacteriophages).
- Phages with high abundance and narrow distribution (high coefficients of variation between metagenomes). This pattern means very high abundance in only a few metagenomes, but partial genome representation. This group mostly consists of the ssDNA *Microviridae* and is further divided into the gokushoviruses (such as the *Chlamydia* phages, which had relatively high percent sequence coverage) and the true microviruses (such as phiX174, which had low percent sequence coverage) (Labonte and Suttle, 2013).
- Phages with low abundance but wide distribution (e.g., some *Pseudomonas* phages (phiKZ, phage 201phi 2-1, and phage EL). This pattern suggests a wide distribution of some highly conserved genes or modules within those phages (Group L in Figure 4).
- Phages with low abundance but high percent sequence coverage (e.g., *Pseudomonas* phage MP38, *Pseudomonas* phage MP29, *Pseudomonas* phage MP22, and Bacteriophage D13 112). These are referred to as Group M in Figure 4.
- Rare phages (e.g., some *Vibrio* phages of the ssDNA phage class *Inoviridae* such as: phages VEJphi, VGJphi, VSK, KSF-1phi, and O139 fs1).

In summary, the metrics were particularly useful in determining outliers or extreme phage groups (e.g., microviruses, cyanoviruses, tectiviruses, etc…). The analysis highlighted the scarcity of sequences shared with RNA phages, the massive yet uneven observed abundance of microviral sequences, and the dominance of T4-like cyanophages and Cooper-like mycobacteriophages in currently sequenced metagenomes.

## Discussion

Estimating phage diversity in nature has generally been more difficult than estimating the diversity of cellular microorganisms—whether by culture-based or molecular methods. This difficulty is, in part, caused by the lack of a set of universal genes common to all phages that can be used for phylogenetic profiling, as opposed to ribosomal DNA and tRNA synthetase genes in cellular life forms (i.e., domains: Archaea, Eubacteria, and Eukartyota). Thus, the emergence of metagenomics has been particularly useful for phage biologists by providing a method for surveying complete phage communities (Breitbart et al., 2002; Angly et al., 2005, 2009; Edwards and Rohwer, 2005). One particularly interesting aspect of these analyses has been the realization that the majority of viral metagenomic sequences do not have any similarities to the databases, highlighting the large amount of “viral dark matter” in the universe. However, the distribution of sequences similar to completely sequenced phage genomes provides important information about the distribution of these representative, well-characterized phages in natural systems. Whereas, early metagenomic studies were highly descriptive in nature, the phenomenal accumulation of metagenomic data now enables researchers to advance from cataloging phage species and functional categories to addressing fundamental questions about phage ecology, evolution, and phage-host co-occurrence and co-evolution. Such questions require the establishment of methods and metrics beyond simply counting metagenomic sequence reads recruited to a phage or taxonomic binning.

In this study, we expand the available analyses for examining phage distributions in unassembled metagenomes by adapting metrics to quantify not only fragment and gene counts, but also (i) coverage density, depth, uniformity, and breadth of phage sequence distribution in metagenomic data sets; and (ii) extent of variability of sequence recruitment to a given phage genomic scaffold. These metrics allowed us to separate phages into groups that more accurately reflect their ecology, which will allow the examination of phage-habitat and phage-host associations in future studies as a wider range of metagenomes are sequenced.

The present work did not aim at developing novel statistical functions or mathematical equations, but rather adapted well-established functions and, sometimes, repurposed metrics used in other fields or applications (such as evenness and kurtosis). The following attributes distinguish the set of metrics that were implemented:

- ***Multiple-level normalization:*** Counting sequence similarity hits is probably the most straightforward and most popular indicator of the abundance of genes and genomes in an ecosystem. With the availability of multiple data sets with different sequence depths and variable read lengths, it has become common practice to normalize the number of hits to the metagenome size (expressed as the number of reads or preferably in as the number of base pairs). Moreover, since a metagenomic data set is just a sample of all the DNA in an environment, any gene (or genome) is more likely to be represented in that sample if it is: (i) more abundant or (ii) larger in size (number of base pairs). Thus, we also normalized hit counts to the length of the gene or genome to which they recruited. The concept of length-normalization is often used in RNA-Seq analysis (Lee et al., 2011) and was introduced by Angly and coworkers in the GAAS suite for estimating relative abundances of full-length phages (Angly et al., 2009). Here, we adopted and expanded length normalization for every analyzed entity (whether it's a protein-encoding gene, genome, or a genomic fragment).
- ***Estimation of coverage density and uniformity:*** Because phage genomes are known for their high mosaicism and because they often contain protein-encoding genes with a wide range of conservation and so-called metagenomic islands (Pasic et al., 2009; Mizuno et al., 2014), we deployed metrics to assess the uniformity vs. variability patterns of coverage plots. For this we describe three different parameters: (i) density or depth, (ii) uniformity or evenness, and (iii) regularity or peakiness. To measure density, we adopted the commonly used measure of number of hits per nucleotide, or the normalized area under the curve (AUC/nNuc) of a coverage plot. To describe coverage uniformity, we used both the coefficient of variation (CV) as an estimator of the *spread* of a coverage plot and the Shannon Evenness metric (E) as an estimator of gene coverage *evenness* in a given genome. Finally, we adopted the *kurtosis* metric that is used to describe distribution curves or line graphs as an estimator of the regularity/irregularity of peaks in a coverage plot.
- ***Multidimensional analysis***. Each of the developed metrics utilized has different strengths and weaknesses. Under specific conditions, some metrics may be more informative than others; some of them may partly correlate; and some could be redundant in certain conditions (e.g., highly abundant and uniformly covered phage genomes will have similar median coverage density and evenness). To take advantage of all the information provided by the different metrics without being misled by one or two of them, we used PCA analysis, which effectively split phages into groups reflecting both their sequence similarity and their ecological distribution.

### Potential limitations and suggested solutions

For some specific phage groups, such as T4-like phages and microviruses, assigning a phage genome was quite difficult. For example, the apparent prevalence of non-marine T4-like phages in most samples may be a result of the overabundance of their closely related cyanophage T4-like genes. In support of this interpretation is the observation that the distribution pattern of phage T4 genome was overshadowed by that of the T4-like cyanophage, P-SSM2 (Ignacio-Espinoza and Sullivan, 2012), especially in ecosystems in which T4-like cyanophages were abundant. In such cases, coverage metrics are crucial in determining whether an entire phage is present in a particular ecosystem, or if the distribution more likely results from conserved genes.

A more striking example is ssDNA phages. Microviruses are ssDNA phages that have previously been shown to be quite abundant in certain metagenomes, especially those created using rolling circle amplification with the phi29 polymerase (e.g., Desnues et al., 2008; Lopez-Bueno et al., 2009; Tucker et al., 2011). Currently sequenced *Microviridae* include the gokushoviruses, which infect obligate intracellular parasites such as *Chlamydia, Spiroplasma*, and *Bdellovibrio*, and the true microviruses (such as phiX174) that infect enteric bacteria (Labonte and Suttle, 2013). However, examining the coverage patterns reveals that most metagenomic sequence reads that match the true microviruses are similar to a tiny fraction of the genome, while the gokushoviruses are frequently covered at nearly 70% (Figure 2B). This pattern of coverage suggests that ssDNA viruses similar to the gokushoviruses are present in the environments examined, while the true microviruses are likely not present. This is an important distinction since simple measurements of abundances would likely miss that distinction, suggesting an abundance of both groups. Another important revelation of this analysis is the confirmation that microviruses were only identified in a limited nMGs, which were amplified using phi29 polymerase, which is known to disproportionately amplify small, circular, ssDNA genomes (Kim and Bae, 2011). However, since the methods used for constructing and sequencing the other metagenomes may have excluded ssDNA viruses, the actual presence or abundance of gokushoviruses in other environments remains unknown. In either case, the relative abundance of these genomes, in particular, is not thought to reflect their natural occurrence.

Finally, in the data sets described here (Table S2), most phages had less than 75% overall sequence coverage per genome (68% of dsDNA phages and 85% of non dsDNA phages were <75% covered). While sequencing depth is a major factor controlling coverage—especially in the case of rare phages, another reason behind this low coverage is that sampled phage genomes may be only partly similar to those in databases while they have other unique, yet-to-be-sequenced modules. This is a limitation that can be addressed through assembling metagenomes, and will likely be reduced as more phages are sequenced and publicly deposited.

### Portability and reproducibility of the methods

The metrics described above are intended to be platform-independent, i.e., they can be applied to any metagenomic analysis pipeline that generates recruitment plots or that map metagenomic hits to a sequence contig/scaffold. The metagenome-level metrics (Table 1) and inter-phage metrics (Table 2) can be applied to any metagenome vs. phage data matrix, where the number of metagenomic reads per phage is calculated at a given *E*-value threshold. The coverage metrics (intra-phage properties, Table 3) can be generated from any recruitment plot where metagenomic sequences are mapped to a phage scaffold or contig. Although we used tBlastX output for mapping, we believe that any other similarity search or mapping tool can be used as well.

Of course, the key to reproducibility in any such analysis is to use the same database/reference set for all comparisons, i.e., the same set of phage genomes has to be used for analyzing all metagenomic data sets, if the results are to be compared to one another. If more phage genomes are added to the Blast database, for example, then any older analyses have to be repeated against the updated database. This is true for any (meta)genomic annotation or analysis pipeline.

## Conclusion

In conclusion, we expanded the existing repertoire of viral metagenomic analysis tools by implementing an array of metrics to describe different aspects of the ecological distribution of archaeal viruses, phages and phage-like sequences in metagenomic data sets. Some of these metrics have been well-developed and efficiently used in phage metagenomic bioinformatics, while others have been used for the first time in this study or adopted from other mathematical and statistical applications and repurposed toward phage analysis. Together, this suite of metrics is useful in expressing different dimensions of phage abundance, extent and breadth of distribution, as well as phage sequence coverage depth and uniformity in diverse ecosystems. The combination of these metrics successfully separates phages in ecologically meaningful ways, which will enable researchers to generate and test biological hypotheses regarding phage ecology and evolution.

## Author contributions

Conceived and designed the study: RA, MB, RE. Developed and applied the methods: RA. Designed tools and wrote scripts: RA, SA, RE. Performed the experiments: RA, BD, SA, RE. Analyzed the data: RA, BD, SA, MB. Wrote the paper: RA, BD, MB, RE.

### Conflict of interest statement

The authors declare that the research was conducted in the absence of any commercial or financial relationships that could be construed as a potential conflict of interest.

## Supplementary material

Figure S1 **Summary statistics of the phage genomes and metagenomic libraries used. (A)** Metagenomic samples classified and sorted by their environments; **(B)** Phages classified by the bacterial families they infect; **(C)** Phages grouped into taxonomic classes.

Table S1 **Raw data calculated for each metagenomic sample used in the study, together with relevant metadata for each metagenome (e.g., biome, environment, size in base pairs, number of reads, GC%)**.

Table S2 **Raw data for all phages, their metadata, and all analysis metrics applied to them**.

Both Tables S1 and S2 are also available online (URL: http://www.phantome.org/eco-locator/v1_tables). The online tables are updatable (e.g., in the case of a change of phage name or the availability of more accurate metagenomic metadata).
