# Supplementary figures and images for: Multidimensional metrics for estimating phage abundance, distribution, gene density, and sequence coverage in metagenomes
Source: Front Microbiol. 2015 May 8;6:381. doi: 10.3389/fmicb.2015.00381 (PMC4424905; doi:10.3389/fmicb.2015.00381)

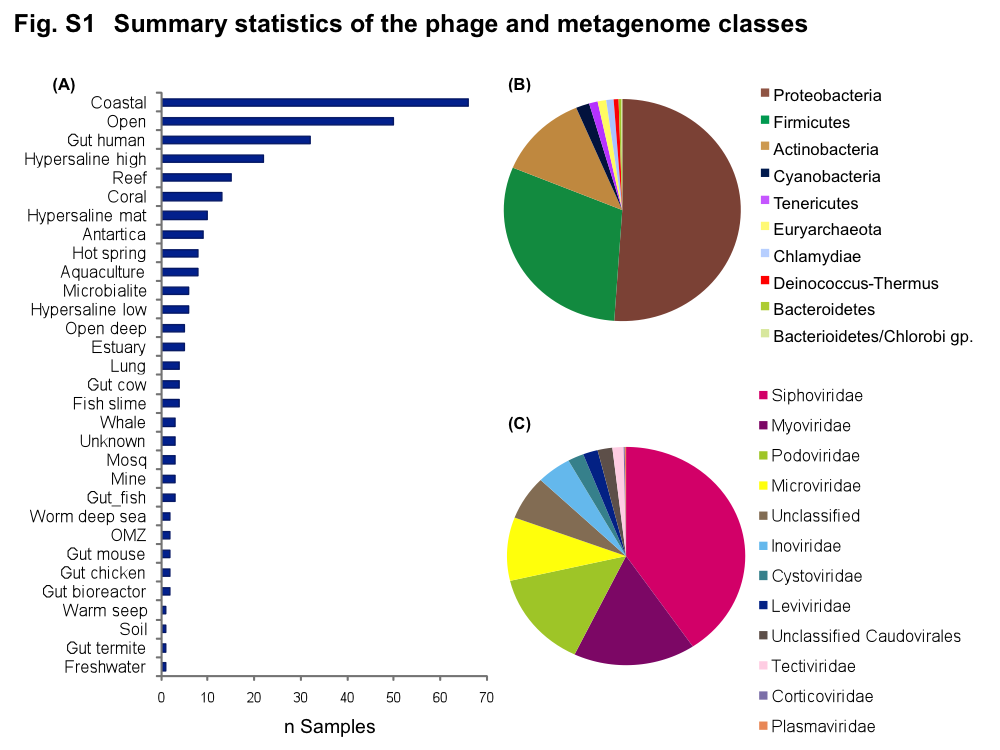

Supplement: Figure S1 — Summary statistics of the phage genomes and metagenomic libraries used. (A) Metagenomic samples classified and sorted by their environments; (B) Phages classified by the bacterial families they infect; (C) Phages grouped into taxonomic classes. [file Image1.TIF]
